# Endocrine Characteristics and Obstetric Outcomes of PCOS Patients with Successful IVF and Non-IVF Pregnancies

**DOI:** 10.3390/jcm13185602

**Published:** 2024-09-21

**Authors:** Mónika Orosz, Fanni Borics, Dávid Rátonyi, Zoárd Tibor Krasznai, Beáta Vida, Tünde Herman, Szilvia Csehely, Attila Jakab, Luca Lukács, Rudolf Lampé, Tamás Deli

**Affiliations:** 1 Department of Obstetrics and Gynaecology, Faculty of Medicine, University of Debrecen, Nagyerdei krt. 98, 4032 Debrecen, Hungarykrasznai.zoard@med.unideb.hu (Z.T.K.); csehely.szilvia@med.unideb.hu (S.C.); lampe.rudolf@med.unideb.hu (R.L.);; 2 Department of Internal Medicine, Faculty of Medicine, University of Debrecen, Nagyerdei krt. 98, 4032 Debrecen, Hungary; borics.fanni@med.unideb.hu; 3 Assisted Reproduction Centre, Clinical Centre, University of Debrecen, Egyetem Tér 1, Nagyerdei krt. 98, 4032 Debrecen, Hungary

**Keywords:** polycystic ovary syndrome, pregnancy complications, in vitro fertilization, pre-pregnancy hormone levels, obstetric outcomes, androgen levels

## Abstract

**Background/Objective:** Infertility affects an estimated 40–50% of women with polycystic ovary syndrome (PCOS), the leading cause of anovulatory infertility, but only a small proportion of the patients require in vitro fertilization (IVF) therapy. Both PCOS and IVF are associated with an increased risk of obstetric complications. To compare preconception endocrine profiles and symptoms, as well as obstetric outcomes of PCOS patients who achieved successful pregnancies with and without IVF treatment. **Methods:** A single-center retrospective cohort study was conducted. Data spanning from 2012 to 2019 were compiled from patients with PCOS who visited the Gynecologic Endocrinology Unit and the Infertility Unit at the Department of Obstetrics and Gynecology, University of Debrecen. Patients diagnosed with PCOS who had had at least one successful delivery beyond the 23rd gestational week at the department were eligible for inclusion in the study. **Results:** Fifteen percent of the 206 pregnancies leading to successful deliveries of 232 newborns in our cohort conceived with IVF. A one year increase in the maternal age increased the odds of being in the IVF group by 22% (OR: 1.222, 95% confidence interval, CI: 1.11–1.35, *p* < 0.001). Baseline DHEAS and androstenedione levels were significantly lower in the IVF group as compared to the non-IVF group: 1 μmol/L increase in the DHEAS level decreased the odds of being in the IVF group by 18% (OR: 0.82, 95% CI: 0.66–1.01, *p* = 0.06), and 1 μg/L increase in the serum androstenedione concentration decreased the same odds by 42% (OR: 0.58, 95% CI: 0.33–1.02, *p* = 0.056). DHEAS levels <6.5 μmol/L had an OR 3.86 (95% CI 1.10–13.50, *p* = 0.04) and LH/FSH ratio <1.3 had an OR 3.58 (95% CI 1.18–10.81, *p* = 0.03) for being in the IVF group. The birth weight (3069 ± 683 g vs. 3362 ± 638 g, *p* = 0.02) and the gestational age (37.23 ± 2.55 vs. 38.54 ± 2.28 weeks, *p* = 0.004) were significantly lower in the IVF group, but in the singleton subgroups, no significant differences could be found. Birth weight percentiles showed no significant difference in either subgroup. In the IVF group, both preterm delivery (29% vs. 8.3%, OR 4.53, 95% CI 1.75–11.70, *p* = 0.002; singleton subgroup: 17.4% vs. 6.3%, OR 3.12, 95% CI 0.89–10.92, *p* = 0.07) and cesarean section (71% vs. 43.2%, OR 3.22, 95% CI 1.40–7.40, *p* = 0.006; singleton subgroup: 65.2% vs. 42.4%, OR 2.55, 95% CI 1.02–6.35, *p* = 0.04) were more frequent than in the non-IVF group. Gestational diabetes and preeclampsia were not significantly different in the IVF and non-IVF groups. **Conclusions:** In PCOS patients with successful pregnancies, those who conceive with IVF seem to be different in their baseline hormone levels and symptoms from the non-IVF group. Adverse obstetric outcomes are more common in the IVF group, and some of these differences persist when adjusting for singleton pregnancies and maternal age, too.

## 1. Introduction

Polycystic ovary syndrome (PCOS) is a heterogeneous condition, recognized as the most prevalent female endocrinopathy among women of reproductive age, as well as a leading cause of anovulatory infertility. The prevalence of PCOS is estimated to range from 10% to 13% [1]. This complex endocrine and metabolic disorder is characterized by hyperandrogenemia, which can present as clinical or laboratory abnormalities, polycystic ovarian morphology, and chronic amenorrhea or anovulation [2,3]. In most cases, PCOS is associated with various metabolic abnormalities, including obesity, hyperinsulinemia, insulin resistance, dyslipidemia, and hypertension. These metabolic disturbances significantly increase the risk of cardiovascular disease, type 2 diabetes mellitus, and metabolic syndrome [4].

Reproductive issues in women with PCOS are diverse, ranging from anovulatory cycles and fertility problems to infertility. The presence of PCOS is associated with an increased risk of pregnancy complications, which may be attributed to hyperandrogenism, insulin resistance, and a higher prevalence of metabolic disorders. These factors contribute to an elevated risk of obstetric and neonatal complications. Women with PCOS also exhibit a significantly increased risk of miscarriage [5] with spontaneous miscarriage rates reported to be 20–40% higher than those in the general population [6,7]. Obesity, hyperinsulinemia, elevated luteinizing hormone concentrations, and endometrial dysfunction, which are prevalent in patients with PCOS, may contribute to the increased risk of early pregnancy loss [8]. The incidence of gestational diabetes mellitus (GDM) can be as much as three times higher in women with PCOS [9]. Additionally, pregnancy-induced hypertension (PIH) and preeclampsia occur three to four times more frequently in this population [10]. The rate of preterm birth is twice as high compared to the healthy population [10] and there is an increased prevalence of small for gestational age (SGA) newborns [6]. Moreover, women with PCOS have higher rates of caesarean deliveries [11].

Infertility affects an estimated 40–50% of women with polycystic ovary syndrome (PCOS) [12]. In cases of anovulatory infertility, PCOS is implicated as the cause in approximately 80% of instances [13,14]. Previous recommendations have suggested that lifestyle modifications combined with metformin treatment may be sufficient for addressing insulin resistance in women with polycystic ovary syndrome (PCOS) who intend to conceive [15]. However, current evidence indicates that metformin alone has a limited role in the treatment of infertility, and patients should be informed that more effective ovulation-inducing drugs are available [16]. Although the dose of metformin in the treatment of PCOS-related symptoms is not well determined, some studies have demonstrated the need for dose-adjustment according to BMI [17]. A higher body mass index (BMI) is associated with lower cumulative pregnancy rates [18]. In obese PCOS patients, a 5–10% reduction in body weight may be effective in achieving ovulation [19]. The primary goal is to restore physiological unifollicular ovulation to reduce the incidence of multiple pregnancies. For those intending to conceive, ovulation induction with medications such as clomiphene citrate, letrozole, or gonadotropins, or surgical treatment such as ovarian drilling, is recommended.

According to recent recommendations [20], letrozole is recommended as the first-line treatment for women with anovulatory infertility. However, in many countries, letrozole is off-label for this indication. As an aromatase inhibitor, letrozole has minimal anti-estrogenic effects on the endometrium and is associated with lower rates of twin pregnancies compared to other ovulation induction agents [21]. Clomiphene citrate, alone or in combination with metformin, may also be effective in inducing ovulation [15]. Neither letrozole nor clomiphene citrate increases the incidence of fetal malformations [22]. If these treatments fail, ovulation induction with gonadotropins can be performed. However, a low-dose, step-up protocol is essential to achieve monofollicular ovulation and avoid ovarian hyperstimulation syndrome (OHSS) [23].

If these treatments are unsuccessful, in vitro fertilization (IVF) or intracytoplasmic sperm injection (ICSI) can be utilized following the failure of primary and secondary ovulation induction therapies. Due to the high number of antral follicles in women with PCOS, the risk of ovarian hyperstimulation syndrome (OHSS) and multiple pregnancies is elevated with IVF treatments. To minimize the likelihood of twin pregnancies and OHSS, single embryo transfer or freeze-all strategy can be recommended [20,24]. It is clear that IVF exposes women to the extra risk of IVF interventions, OHSS, and twin pregnancies, and is therefore best avoided if not inevitable. Assisted reproductive techniques in general, and IVF in particular, are associated with both fetal/neonatal and maternal pregnancy-associated risks. These include early pregnancy loss, multiple gestation incidence and related risks, low birth weight, hypertensive disorders and gestational diabetes, spontaneous preterm birth, abnormal placentation, increased maternal mortality, and severe maternal morbidity (postpartum hemorrhage, sepsis, ICU admission). The precise reasons for this increase in adverse outcomes are not clear, but potential candidates include maternal and paternal characteristics, underlying medical conditions associated with subfertility and infertility, sperm factors, the use of fertility medications, laboratory conditions during embryo culture, culture medium, cryopreservation and thawing, prenatal genetic testing and embryo biopsy, differences in obstetric management, an increased proportion of multiple gestations and vanishing twins, or a combination of these factors [25]. Due to the increased risk of multifetal gestation and its related complications during IVF, to have a good estimation of fetal risks from the beginning of pregnancy, early and precise determination of zygosity, chorionicity, and amnionicity is inevitable [26].

Despite these well-known risks associated with IVF pregnancies, it has not been determined what the major endocrine differences are in PCOS patients who finally require IVF for a successful pregnancy as compared to those that do not, as well as what the differences in obstetric outcomes and complications are in the IVF and non-IVF groups of these patients. In our retrospective study, we aimed to determine these differences in the IVF and non-IVF conceived PCOS groups.

## 2. Materials and Methods

With the permission of the Ethics Committee of the University of Debrecen, electronic patient records of all patients with PCOS who attended the Gynecologic Endocrinology Unit and the Infertility Unit at the Department of Obstetrics and Gynaecology, University of Debrecen between 2012 and 2019 were searched. A number of 738 PCOS patients were identified and their medical records retrieved. From this group, all patients diagnosed with PCOS, and having had at least one successful delivery beyond gestational week 23 at our department, were included in the study. Thus, the study cohort consisted of 160 patients diagnosed with PCOS, each of whom had at least one viable delivery, resulting in a total of 206 pregnancies and 232 newborns. All patients received antenatal care and delivered at our department, allowing the collection of obstetric and neonatal data. PCOS was diagnosed according to the Rotterdam criteria [27]. Other endocrinological diseases with similar symptoms including non-classical congenital adrenal hyperplasia (NCAH), hyperprolactinemia, Cushing’s disease, and thyroid disorders were excluded in each case by routinely testing 17-OH-progesterone, prolactine, cortisol, and TSH, in accordance with the Rotterdam criteria. IVF treatment was chosen as a third-line therapy in accordance with international guidelines for the treatment of PCOS-related infertility [20] if endocrine-metabolic therapy, ovulation induction treatment, or insemination attempts for 3–6 cycles were unsuccessful, or if other indications for IVF were present (andrologic, tubal, other).

Clinical, laboratory, and obstetric data were retrospectively collected from the E-MedSolution electronic medical database system. Baseline preconception serum hormone concentrations including gynecological hormones (cycle day 3, i.e., CD3 FSH, LH, E2, and cycle day 21–23, i.e., CD23 progesterone), androgens (testosterone, androstenedione, dehydroepiandrosterone sulfate), sex hormone-binding globulin (SHBG), prolactin, TSH and 25-hydroxy-vitamin D levels, were also documented. Not all hormone levels were available in the case of all patients, given that in some referred cases, they had had the laboratory examinations made elsewhere, they were not mentioned in the documentation and were not possible to track down, or certain laboratory tests were not carried out as no therapeutic consequence was expected from the results. However, all patients who had a given test available was included in the study. Baseline endocrine symptoms assessed included hyperandrogenism, irregular cycles, insulin resistance, and known andrologic abnormality (as defined in the 2010 WHO guidelines [28]), whereas preconception endocrine treatments analyzed were thyroxine substitution and metformin therapy. Insulin resistance was diagnosed if any of the following was found on standard 75 g oral glucose tolerance testing (OGTT): impaired fasting glucose, IFG (fasting serum glucose 6.1–6.9 mM), impaired glucose tolerance, IGT (120 min serum glucose ≥7.8 mM), fasting serum insulin >22 mU/L, 120 min serum insulin >80 mU/L, HOMA-IR index >2.5, 120 min serum insulin >60 min serum insulin. Obstetric outcomes included the mode of delivery (vaginal birth or caesarean section), newborn gender, and pregnancy complications such as gestational diabetes, preeclampsia, and preterm delivery.

Laboratory tests: Serum thyroid stimulating hormone (TSH), dehydroepiandrosterone sulfate (DHEA-S), sex hormone binding globulin (SHBG), and testosterone (T) were measured using electrochemiluminiscence (ECLIA) immunoassay (Roche Diagnostics GmbH, Mannheim, Germany). Serum androstenedione was measured by using chemiluminescence (CLIA) immunoassay (DiaSorin Inc., Stillwater, MN, USA).

### Statistical Analysis

Statistical analysis was performed using the IBM SPSS Statistics 25 software. Normality assumptions were assessed using the Kolmogorov–Smirnov test. Levene’s test was used to determine equality of variances. For parametric variables, independent sample *t*-test was used to compare equality of means, and Pearson’s correlation to determine the degree of association, while for non-parametric variables, Spearman’s correlation for association and Mann–Whitney U test for comparison of means or medians were used. Nominal variables were compared using the Chi-square test or Fisher’s Exact test. Binary and multinomial logistic regression analysis was applied to determine odds ratios and adjusted odds ratios and 95% confidence intervals (95% C.I.). A p value less than 0.05 was considered statistically significant. Data are shown as mean ± SD or median and interquartile range, where applicable.

## 3. Results

A number of 738 patients with PCOS attended the Gynecologic Endocrinology and the Infertility Units in the given period of time. Out of them, 160 patients with 206 pregnancies sought antenatal care at our institution and subsequently delivered 232 newborns.

**Figure 1:**
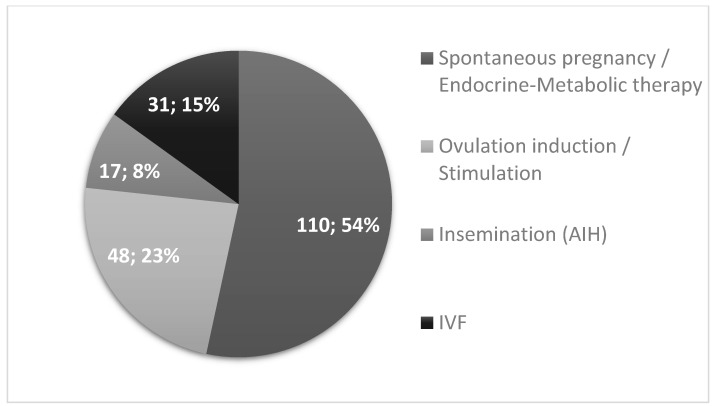
Distribution of fertility treatments among PCOS patients with successful pregnancies. (AIH—artificial insemination, IVF—in vitro fertilization).

Pregnancies were stratified based on the method of conception. Notably, 46% of the patients achieved pregnancy through Assisted Reproductive Techniques (ART), including ovulation induction (23%), intrauterine insemination (8%), and in vitro fertilization (15%) (Figure 1). 

The remaining 54% managed to become pregnant spontaneously or using metabolic-endocrine therapy and lifestyle modification, but excluding direct ovulation induction. Andrologic abnormality (male factor) was found in seven (22%) patients undergoing IVF. Among non-IVF patients, eight individuals were diagnosed with andrological disorders (4%), but in this group, semen analysis was only carried out in the partners of some of the patients receiving ovulation induction therapy and in the partners of those participating in IUI.

We performed a comparative assessment of pre-conception baseline endocrine profiles, maternal age, gestational duration, birth weight, and birth weight percentile distribution between the IVF and non-IVF groups (Table 1).

Our analyses revealed no statistically significant differences in preconception follicle-stimulating hormone (FSH), luteinizing hormone (LH), and estradiol (E2) concentrations. Nevertheless, progesterone levels during the luteal phase exhibited a trend toward statistical significance, being higher in the IVF group (9.83 ± 10.00 vs. 5.63 ± 7.39, *p* = 0.07). Furthermore, no discernible distinctions were observed between the two groups in testosterone and sex hormone-binding globulin (SHBG) levels. Conversely, androstenedione (2.20 ± 1.11 vs. 3.16 ± 1.66, *p* = 0.05) and dehydroepiandrosterone sulfate (DHEAS) levels (6.11 ± 3.13 vs. 8.09 ± 3.37, *p* = 0.05) were significantly lower in the IVF group compared to the non-IVF group.

Maternal age was significantly higher in the group that subsequently needed IVF interventions (all pregnancies: 33.58 ± 4.36 vs. 29.83 ± 4.38, *p* < 0.001; singleton pregnancies: 33.57 ± 4.26 vs. 29.94 ± 4.26, *p* < 0.001) (Table 1 and Table 2).

Our investigation revealed non-significant correlations between preconception endocrinological symptoms and therapeutic interventions (hyperandrogenism, hyperandrogenism, history of miscarriage, insulin resistance, and metformin use or thyroxine substitution) in forecasting subsequent need for in vitro fertilization (IVF) (Table 3). However, menstrual irregularity trended to increase the odds of having IVF later on.

Moreover, no significant difference was seen in the body mass index (BMI) of the IVF and non-IVF group. Similarly, levels of vitamin D did not exhibit statistically significant differences between the IVF and non-IVF groups (Table 1).

We conducted univariable logistic regression analyses to evaluate the odds ratios (ORs) associated with the subsequent necessity for in vitro fertilization (IVF). Our findings revealed that each one-year increment in maternal age augmented the likelihood of requiring IVF by 22% (OR: 1.222, 95% confidence interval [CI]: 1.11–1.35, *p* < 0.001). Conversely, a one-unit (μmol/L) increase in dehydroepiandrosterone sulfate (DHEAS) levels corresponded to an 18% reduction in the likelihood of necessitating IVF (OR: 0.82, 95% CI: 0.66–1.01, *p* = 0.06), while a one-unit (μg/L) increase in androstenedione levels was associated with a 42% decrease in the odds of IVF requirement (OR: 0.58, 95% CI: 0.33–1.02, *p* = 0.056) (Table 4).

**Figure 2:**
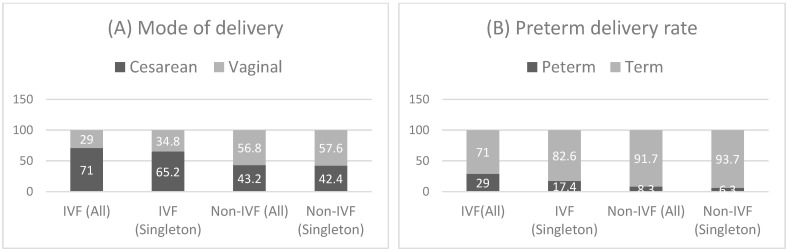
Mode of delivery (**A**) and preterm delivery rate (**B**) among IVF and non-IVF pregnancies in the groups of all and singleton pregnancies. (IVF—in vitro fertilization).

In pregnancies conceived via IVF, both singleton and twin gestations exhibited markedly elevated incidences of preterm delivery (all pregnancies: OR 4.53, 95% CI: 1.75–11.70, *p* = 0.002; singleton pregnancies: OR 3.12, 95% CI: 0.89–10.92, *p* = 0.07) and cesarean section (all pregnancies OR 3.22, 95% CI: 1.4–7.4, *p* = 0.006; singleton pregnancies: OR 2.55, 95% CI: 1.02–6.35, *p* = 0.04). These differences persisted after adjusting for maternal age: preterm delivery (aOR 5.06, 95% CI 1.81–14.18, *p* = 0.002); singleton subgroup: aOR 3.38, 95% CI 0.88–12.88, *p* = 0.075) and cesarean section (aOR 2.82, 95% CI 1.19–6.71, *p* = 0.019; singleton subgroup: aOR 2.25, 95% CI 0.87–5.81, *p* = 0.09) both had an increased risk in the IVF group, although in the singleton subgroup, these elevated odds ratios just missed statistical significance. On the contrary, no significant disparities were observed in the incidence rates of gestational diabetes and preeclampsia between the IVF and non-IVF cohorts (Table 5 and Table 6, Figure 2).

Regarding newborn birth weight outcomes, significant difference was detected only among the whole group of pregnancies (3069 ± 683 vs. 3362 ± 638, *p* = 0.02) (Table 1). Notably, the presence of twins within the IVF group might have been responsible for reduced birth weights, as no significant difference was evident between the IVF and non-IVF singleton subgroups (3289.13 ± 496.08 vs. 3376.62 ± 628.71, *p* = 0.52) (Table 2). Furthermore, there were no differences in birth weight percentiles between the IVF and non-IVF cohorts (all pregnancies: 51.37 ± 33.67 vs. 54.85 ± 29.82, *p* = 0.56; singleton pregnancies: 54.09 ± 32.63 vs. 55.11 ± 29.83, *p* = 0.88) (Table 1 and Table 2).

## 4. Discussion

To the best of our knowledge, this is the first study comparing preconception endocrine parameters, symptoms, and treatments, as well as obstetric outcomes in PCOS patients that had successful pregnancies following IVF treatment or conceiving without IVF. Fifteen percent of the 206 pregnancies leading to successful deliveries in our cohort conceived with IVF. Fifty-four percent of the patients could become pregnant after endocrine and metabolic treatment of the abnormalities associated with PCOS, whereas the remaining 31% needed ovulation induction therapy, insemination, or the combination of the two. It is widely recognized that IVF in PCOS is best avoided, if possible, as the interventions during the procedure itself, OHSS, and twin pregnancies pose extra risk to the patients. Furthermore, IVF is far more expensive than the other treatment options. Outside of all these negative effects of IVF, it can be associated with adverse obstetric outcomes, but the IVF and non-IVF PCOS pregnancy groups have not been directly compared.

Comparing preconception hormone levels, FSH and LH were not significantly different between the IVF and non-IVF groups, but the LH/FSH ratio trended to be higher in the non-IVF group (Table 1). Based on this, we dichotomized the LH/FSH data, and if we determined a threshold at LH/FSH = 1.3, we found that the odds of being in the successful IVF pregnancy group instead of the successful non-IVF pregnancy group was 3.58 (95% C.I. 1.18–10.81). We also found that the luteal phase progesterone level was nearly significantly higher in the IVF group at baseline (almost double, 9.83 vs. 5.63 μg/L, Table 1). This may mean that a worse ovulatory function was present in the non-IVF group, making ovulation induction strategies reasonable for them, resulting in pregnancy before IVF would have become necessary.

Baseline androgen levels were also different in the IVF and non-IVF groups. Testosterone levels were not different, but both androstenedione and DHEAS levels were significantly lower in the IVF subgroup (Table 1). Actually, 1 μmol/L increase in the DHEAS level decreased the odds of being in the IVF group by 18%, and 1 μg/L increase in the serum androstenedione concentration decreased the same odds by 42% (Table 4). DHEAS levels below 6.5 μmol/L resulted in a 3.86-fold increase in the odds for being in the IVF group. The differences in androgen levels could be explained by the fact that the IVF population has a significantly higher age, and age is known to correlate with DHEAS levels. DHEAS levels keep decreasing after young adulthood in both males and females [29], and this has been described in PCOS populations, too [30]. Nevertheless, the 25–30% difference in the serum DHEAS levels between the IVF and non-IVF groups (6.1 and 8.1 μmol/L, respectively, see Table 1) cannot be explained by the 3.5-year average age difference between them, because the average yearly drop in DHEAS levels is only 2–3% starting at the age of 20 to 30 in the general population [31]. During IVF treatment, DHEA is widely used to improve outcomes in poor responders and those with diminished ovarian reserve, and several studies and meta-analyses support this routine [32,33], although the question is not completely settled. Androgen concentrations, however, have not been shown clearly to correlate with IVF success [34].

Preconception endocrine symptoms and treatments did not change the odds of being in the IVF group significantly: metformin treatment, thyroxine substitution, hyperandrogenism, the presence of andrologic abnormality or miscarriage in the medical history, or the presence of insulin resistance did not seem to have this kind of predictive value. Although just missing statistical significance, a trend in the presence of irregular cycles halving the odds of having IVF before a successful pregnancy could be observed. This seems logical in the sense that irregular cycles in PCOS mostly indicate oligo-anovulation, which can often be treated successfully with endocrine-metabolic therapy or ovulation induction, deeming IVF unnecessary.

As expected, the age of the IVF group was significantly higher, both among all pregnancies and the singleton subgroup, too (Table 1 and Table 2). A one-year increase in the maternal age increased the odds of being in the IVF group by 22% (Table 4). BMI was not different between the IVF and non-IVF group, both groups being overweight as usual in PCOS populations (Table 1), although BMI data were often not recorded in the source charts. It is worthy of note, however, that the studied cohort was neither too old as compared to the general Hungarian childbearing population or the general IVF population, nor very obese, and thus age or BMI extremities did not distort the results. It is interesting, though, that maternal age > 31 years resulted in a 3.89-fold increase of the odds for being included in the IVF subgroup (Table 3).

Our results demonstrate that IVF conception in PCOS can be associated with adverse obstetric outcomes: we found that in both twin and singleton IVF-conceived pregnancies, preterm delivery and cesarean birth have 2.5 to 4.5-fold greater odds as compared to non-IVF pregnancies, greater odds being present in the twin pregnancy group (Table 5 and Table 6). As maternal age was significantly higher in the IVF group, the differences could be attributed to this, but the differences in preterm delivery and cesarean section rates persisted after adjustment for maternal age. Furthermore, these differences cannot be attributed to either preeclampsia or gestational diabetes, because although both of these pregnancy-associated diseases were more common than in the general population, there was no significant difference in the IVF and non-IVF groups. Our results are in line with former studies: Palomba et al. reported a 3–4-fold increase in the risk of pregnancy induced hypertension and preeclampsia, a three-fold increase in the risk of gestational diabetes, and a two-fold increase risk of premature delivery in the general PCOS population with successful pregnancies [10]. It was also shown, that multiple pregnancies in PCOS increase the risk of pregnancy induced hypertension (OR 2.030), preeclampsia (OR 2.879), gestational diabetes (OR 1.358), preterm delivery (OR 8.466), and cesarean section (OR 5.146) [35]. Among IVF-treated patients, the PCOS group was compared to the non-PCOS infertility subgroup by Sha and colleagues, and similar results were found: the odds of gestational diabetes (OR 2.67), pregnancy induced hypertension (OR 2.06), and preterm birth (OR 1.60) were significantly increased. Yet, none of these studies aimed to compare IVF and non-IVF pregnancies in PCOS [36].

Our data show that deliveries occurred significantly earlier in IVF pregnancies, and even in singletons this trend remained unchanged (Table 5 and Table 6). However, the birth weight was smaller in the IVF group only because of the presence of twins, since no difference was seen between singleton subgroups. More importantly, IVF or non-IVF conception did not seem to make a difference in the occurrence of small for gestational age (SGA) or intrauterine growth restriction (IUGR), as birth-weight percentiles were similar in the two groups regarding both all newborns and the singleton subgroup. In none of our subgroups did we see IUGR, as the birth weight percentiles were a little above 50%, rather than below it. Former data about PCOS is conflicting about IUGR being more common in PCOS. One study found a significant 2.62-fold greater odds of IUGR in PCOS [37], whereas others did not find a significant difference [38]. But, again, none of these studies compared the birth weight and birth weight percentiles of the IVF and non-IVF conceived subgroups in PCOS.

Our study has several strengths. We compared IVF and non-IVF conceived successful pregnancies of PCOS patients, whereas most studies in the literature that deal with obstetric and neonatal outcomes compare PCOS populations [10] IVF populations [18] and (rarely) IVF-conceived PCOS populations [39] to the general population. However, the real question in clinical practice is not the risk as compared to the general population, but the extra risk when IVF treatment is chosen, which is provided by our results. Another strength is that we had detailed preconception endocrine laboratory results, clinical symptoms, and endocrine treatments at our disposal, that could be analyzed with regard to the subsequent use or avoidance of IVF. Based on these results and determining some thresholds that were chosen based on the former analysis of the variables (e.g., DHEAS, AD, LH/FHS values), predictors for the need of subsequent IVF, as well as potential treatment options (e.g., DHEA therapy) and targets for future investigations could be identified. The single-centre character of the study is also an advantage in that diagnosis, treatment or intervention principles were certainly uniform. Finally, many of the articles cited in this paper were based on infertility treatments of PCOS patients attending infertility clinics, whereas our patients were an unselected population of PCOS patients treated for infertility and/or other symptoms of PCOS not only in an infertility clinic, but also a reproductive endocrinology clinic, thus allowing broader conclusions and representing better the real-life PCOS population, often seeking medical help first outside the scope of infertility treatments.

The study certainly has the limitation of being retrospective in character, which limited the availability of certain data. Despite the originally large number of PCOS patients whose charts were retrieved, only about 20% of them had successful pregnancies (although many had more than one). This is because not only infertility patients’ charts were searched. Because of the various reasons and symptoms of PCOS that prompted the original diagnostic examinations, several patients did not have all the parameters assessed at baseline. The reason for this is that for example androgen levels were often not tested if the diagnosis could be made without laboratory androgen testing (either because ovulatory disorder + PCO morphology on ultrasonography were present, or because clinical hyperandrogenism made detailed laboratory testing for androgens unnecessary), or the exact androgen levels did not have a treatment-modifying significance (e.g., oral contraceptive initiation was planned in mild hirsutism). Furthermore, some referred patients might have had laboratory tests made elsewhere and detailed results were not available in the documentation. This finally resulted in having small numbers in some subgroups. We are also aware that the two compared groups are heterogenous as far as the cause of subfertility is concerned: IVF group: unsuccessful endocrine and ovulation induction treatments and/or male factor and/or potential tubal factor and/or consideration of higher maternal age at treatment choice and/or presence of other idiopathic factors; non-IVF group: spontaneous pregnancy or ovulation induction therapy or endocrine therapy or intrauterine insemination. Yet, we believe this does not modify the primary aim of the study, which was to determine differences in baseline characteristics or obstetric outcomes between the two groups, because the greatest dichotomy in the treatment of PCOS-related infertility lies between IVF and non-IVF treatments.

In summary, our data seem to indicate that there are significant differences between PCOS patients with successful pregnancies, depending on whether they conceive by IVF or without IVF. The IVF and non-IVF groups have differences in both their preconception endocrine parameters, symptoms, and treatments, as well as the subsequent obstetric risks and outcomes. These results may help to predict the need for IVF treatment in PCOS, and direct obstetric care during pregnancy.

## Figures and Tables

**Table 1:** Comparison of pre-pregnancy hormone levels, maternal age, gestational age, birth weight, and birth weight percentile in the group of all pregnancies of PCOS patients conceived with IVF and non-IVF.

	IVF		Non-IVF		
	Mean ± S.D.	(*n*)	Mean ± S.D.	(*n*)	*p*
Maternal age (y)	33.58 ± 4.36	(31)	29.83 ± 4.38	(174)	**<0.001**
Gestational age (wk)	37.23 ± 2.55	(31)	38.54 ± 2.28	(169)	**0.004**
Birth weight (g)	3069 ± 683	(31)	3362 ± 638	(168)	**0.02**
Birth weight %	51.37 ± 33.67	(31)	54.85 ± 29.82	(168)	0.56
TSH (mU/L)	1.91 ± 0.96	(21)	2.14 ± 1.28	(88)	0.44
E2 (CD3) (ng/L)	49.63 ± 37.55	(21)	53.86 ± 43.17	(75)	0.68
FSH (CD3) (IU/L)	6.43 ± 2.35	(21)	6.11 ± 2.16	(73)	0.56
LH (CD3) (IU/L)	7.74 ± 6.79	(21)	9.82 ± 8.23	(73)	0.29
LH/FSH (CD3)	1.24 ± 1.19	(21)	1.57 ± 0.86	(72)	0.16
PROG (CD21) (μg/L)	9.83 ± 10.00	(15)	5.63 ± 7.39	(60)	**0.07**
PRL (μg/L)	19.82 ± 15.53	(23)	16.30 ± 9.60	(99)	0.17
TEST (nmol/L)	1.44 ± 1.31	(12)	1.61 ± 0.76	(56)	0.55
SHBG (nmol/L)	72.01 ± 54.40	(12)	54.29 ± 37.60	(51)	0.19
AD (μg/L)	2.20 ± 1.11	(13)	3.16 ± 1.66	(58)	**0.05**
DHEAS (μmol/L)	6.11 ± 3.13	(13)	8.09 ± 3.37	(58)	**0.05**
BMI (kg/m^2^)	29.52 ± 4.37	(9)	28.04 ± 6.26	(50)	0.50
25-OH-Vitamin-D (nmol/L)	61.00 ± 6.88	(5)	64.04 ± 25.54	(24)	0.80

(TSH—Thyroid-stimulating hormone, E2—oestradiol, FSH—Follicle-stimulating hormone, LH—luteinizing hormone, Prog—progesterone, PRL—prolactin, Test- testosterone, SHBG—Sex Hormone Binding Globulin, AD—androstenedione, DHEAS—dehydroepiandosterone sulfate, BMI—body mass index).

**Table 2:** Comparison of maternal age, gestational age, birth weight, and birth weight percentile in the group of **singleton pregnancies** of PCOS patients conceived with IVF and non-IVF.

	IVF		Non-IVF		
	Mean ± S.D.	(*n*)	Mean ± S.D.	(*n*)	*p*
Maternal age (y)	33.57 ± 4.26	(23)	29.94 ± 4.26	(163)	**<0.001**
Gestational age (wk)	38.09 ± 1.47	(23)	38.62 ± 2.26	(158)	**0.08**
Birth weight (g)	3289.13 ± 496.08	(23)	3376.62 ± 628.71	(157)	0.52
Birth weight %	54.09 ± 32.63	(23)	55.11 ± 29.83	(154)	0.88

(IVF—in vitro fertilization).

**Table 3:** Odds ratios (OR) for IVF conception, based on pre-pregnancy endocrine symptoms, treatments, and hormonal parameters.

	IVF		Non-IVF				
	*n*	%	*n*	%	OR for IVF	95% C.I.	*p*
**Metformin**							
Yes	7	24.1	35	26.9	0.86	0.34–2.20	0.76
No	22	75.9	95	73.1			
**Thyroxine substitution**							
Yes	7	24.1	22	17.2	1.53	0.58–4.03	0.39
No	22	75.9	106	82.8			
**Hyperandrogenism**							
Yes	6	20.7	42	33.1	0.53	0.20–1.40	0.20
No	23	79.3	85	66.9			
**Irregular cycle**							
Yes	12	41.4	75	58.6	0.50	0.22–1.13	0.10
No	17	58.6	53	41.4			
**Andrologic abnormality**							
Yes	6	66.7	7	36.8	3.43	0.65–18.22	0.15
No	3	33.3	12	63.2			
**Miscarriage in history**							
Yes	7	30.4	29	23.8	1.40	0.53–3.74	0.50
No	16	69.6	93	76.2			
**Insulin resistance**							
Yes	13	43.3	52	30.6	1.74	0.79–3.83	0.17
No	17	56.7	118	69.4			
**Age > 31 y**							
Yes	21	67.7	61	35.1	**3.89**	**1.72–8.79**	**0.001**
No	10	32.3	113	34.9			
**LH/FSH < 1.3**							
Yes	16	76.2	34	47.2	**3.58**	**1.18–10.81**	**0.03**
No	5	33.8	38	52.8			
**AD < 2.5 (μg/L)**							
Yes	7	53.8	24	41.4	1.65	0.49–5.54	0.42
No	6	46.2	34	58.6			
**DHEAS < 6.5 (μmol/L)**							
Yes	8	61.5	17	29.3	**3.86**	**1.10–13.50**	**0.04**
No	5	38.5	41	70.7			

(AD—androstenedione, DHEAS—dehydroepiandrosterone sulfate, IVF—in vitro fertilization, LH—luteinizing hormone, FSH—follicle stimulating hormone).

**Table 4:** Odds ratios (OR) for IVF per one unit increase in age, androgens, and LH/FSH ratio.

	OR for IVF per 1 Unit Change	95% C.I.	*p*
Age (y)	1.222	1.11–1.35	**<0.001**
DHEAS (μmol/L)	0.82	0.66–1.01	**0.06**
AD (μg/L)	0.58	0.33–1.02	**0.056**
Test. (nmol/L)	0.78	0.35–1.74	0.55
LH/FSH	0.62	0.31–1.22	0.16

(FSH—Follicle-stimulating hormone, LH—luteinizing hormone, Test—testosterone, AD—androstenedione, DHEAS—dehydroepiandrosterone sulfate).

**Table 5:** Odds of obstetric outcomes following IVF among all pregnancies.

	IVF		Non-IVF							
	*n*	(%)	*n*	(%)	OR	95% C.I.	*p*	aOR	95% C.I.	*p*
**Sex of newborn**										
Female	16	(51.6)	78	(46.2)	1.24	0.58–2.68	0.58	1.63	0.72–3.68	0.24
Male	15	(48.4)	91	(53.8)						
**Preterm delivery**										
Yes	9	(29.0)	14	(8.3)	**4.53**	**1.75–11.70**	**0.002**	**5.06**	**1.81–14.18**	**0.002**
No	22	(71.0)	155	(91.7)						
**Cesarean delivery**										
Yes	22	(71.0)	73	(43.2)	**3.22**	**1.40–7.40**	**0.006**	**2.82**	**1.19–6.71**	**0.019**
No	9	(29.0)	96	(56.8)						
**Gestational diabetes**										
Yes	14	(48.3)	60	(46.9)	1.06	0.47–2.37	0.89	0.93	0.38–2.24	0.87
No	15	(51.7)	68	(53.1)						
**Preeclampsia**										
Yes	3	(10.0)	17	(8.6)	1.18	0.32–4.39	0.80	1.22	0.31–4.85	0.77
No	27	(90.0)	149	(91.4)						

(IVF—in vitro fertilization).

**Table 6:** Odds of obstetric outcomes following IVF among singleton pregnancies.

	IVF		Non-IVF							
	*n*	(%)	*n*	(%)	OR	95% C.I.	*p*	aOR	95% C.I.	*p*
**Sex of newborn**										
Female	13	(56.5)	74	(46.8)	1.48	0.61–3.56	0.39	1.96	0.77–4.99	1.16
Male	10	(43.5)	84	(53.2)						
**Preterm delivery**										
Yes	4	(17.4)	10	(6.3)	**3.12**	**0.89–10.92**	**0.07**	**3.38**	**0.88–12.88**	**0.075**
No	19	(82.6)	148	(93.7)						
**Cesarean delivery**										
Yes	15	(65.2)	67	(42.4)	**2.55**	**1.02–6.35**	**0.04**	**2.25**	**0.87–5.81**	**0.09**
No	8	(34.8)	91	(57.6)						
**Gestational diabetes**										
Yes	11	(52.4)	56	(45.9)	1.30	0.51–3.28	0.58	1.13	0.41–3.07	0.81
No	10	(47.6)	66	(54.1)						
**Preeclampsia**										
Yes	3	(13.6)	13	(8.1)	2.38	0.70–8.05	0.16	2.38	0.65–8.66	0.18
No	19	(86.4)	147	(91.9)						

(IVF—in vitro fertilization).

## Data Availability

The data presented in this study are available by contacting the corresponding author.
